# Relationships Among Job Burnout, Generativity Concern, and Subjective Well-Being: A Moderated Mediation Model

**DOI:** 10.3389/fpsyg.2021.613767

**Published:** 2021-02-25

**Authors:** Xingniu Lan, Yinghao Liang, Guirong Wu, Haiying Ye

**Affiliations:** ^1^School of Education and Music, Hezhou University, Hezhou, China; ^2^Department of Educational Science, Sichuan University of Arts and Science, Dazhou, China

**Keywords:** job burnout, subjective well-being, generativity concern, moderated mediation effect, intimacy, adaptability

## Abstract

**Background:** Policemen all over the world are tasked with the heavy work of maintaining social security. With the imbalance in mentality brought about by high population density and social transformation, the work of the Chinese police is particularly hard. As the window of demographic dividend is closing and the number of newborns is insufficient, China has started to adjust its established fertility policy to encourage a family to have two children. However, the results have not met the expectations of the policy adjustment. It is generally believed that factors such as high work pressure, high parenting costs, and low levels of happiness may be the main reasons for low fertility intentions. Studying this typical population of police officers may explore the relationship between work stress, happiness, and reproductive concerns, and provide evidence of Chinese sample.

**Objectives:** To explore the relations between job burnout, subjective well-being, and generativity concern in Chinese police officers.

**Methods:** The study used a cross-sectional survey to collect data from 494 police officers from H city in China. The participants completed the Maslach Burnout Inventory (MBI), the Family Adaptability and Cohesion Scale (FACESII), the Loyola Generativity Scale (LGS), and the Satisfaction with life scale (SWLS). Moderated mediation effect models assessed the association between job burnout, subjective well-being, and generativity concern.

**Results:** Job burnout had a significant negative predictive effect on both subjective well-being and generativity concern, and subjective well-being played a mediating role between job burnout and generativity concern. In addition, family intimacy and adaptability had a significant negative moderating effect between subjective well-being and generativity concern. In a conclusion, there is a moderated mediating effect between job burnout and generativity concern.

**Conclusion:** Subjective well-being played a mediating role between job burnout and generativity concern.

## Introduction

Generativity, “the concern in establishing and guiding the next generation,” is the main task of development in mid-adulthood, and its acquisition and development are included in the activities of nurturing, production, and creation, which are the further development of ego-identity and the creation and extension of self-worth, and the goal of generativity is to promote individual development (Erikson, 1963). Generativity involves both the motive and the behavior to support and guide younger people and to benefit “future generations” and generativity is adaptive, contributing positively to personality development and well-being (Lawford et al., 2005; Doerwald et al., 2020). Building on Erikson's work, Kotre (1984) maintained that generativity is not attached to a certain age *per se*, but rather differently expressed across the adult lifespan. Furthermore, he distinguished four types of generativity: biological (e.g., childbearing), parental (e.g., nurturing children), technical (e.g., passing on skills and knowledge), and cultural generativity (e.g., passing on cultural ideas). With the recent development of China's aging population and the introduction of a nationwide two-child policy, the study of generativity sense, which is important for social and individual development, has received widespread attention from researchers (McKeering and Pakenham, 2000; Clark and Arnold, 2008; Garcia et al., 2018). For example, researchers have found that generativity sense has an impact on individual career development, that fathers tend to benefit more than mothers from child-rearing activities, and that their commitment to family and child-rearing does not conflict with their social affairs and career development and success, but rather enhances men's self-efficacy (McKeering and Pakenham, 2000). Peterson and Duncan (2007) found that generativity sense positively predicts positive female personality traits, marital and maternal satisfaction. As the research progressed, researchers, not content with Erikson's theories that emphasized only the positive dimensions of generativity sense for the middle-aged themselves and the subjects of generativity sense, began to explore the negative dimensions of generativity sense (Kotre, 1984) and developed an integrative theoretical framework of generativity sense (McAdams and de St Aubin, 1992; McAdams et al., 1993). They explained generativity sense as a structural model of transformation that encompasses individual, inter-individual, and social dynamics and includes seven components, of which generativity concern is central. Researchers have found a significant correlation between generativity concern and job burnout (Zacher et al., 2015), but the mechanisms by which this occurs are unclear. Therefore, this study tends to explore the impact of job burnout on generativity concern.

Job burnout is the physical, emotional, behavioral and mental exhaustion that occurs when an individual is unable to cope with the excessive demands of the external world beyond his or her energy and resources (Freudenberger, 1974; Maslach and Jackson, 1981; Shaukat et al., 2017). Job burnout is manifested in three main areas: emotional exhaustion, dehumanization, and decreased personal fulfillment. Researchers have found that job burnout affects an individual's generativity concern (Kooij and Van De Voorde, 2011). Both emotional exhaustion and depersonalization in burnout were significantly correlated with family-work conflict, and excessive family-work conflict significantly reduced an individual's generativity focus (Netemeyer et al., 1996). On the one hand, mid-adulthood is an important period in an individual's career development, with heavy workloads that make the individual more prone to burnout (Rothenberger, 2017; Tawfik et al., 2018; Kowalczuk et al., 2020). While on the other hand, mid-adulthood is also a time when the family is faced with a number of issues, whether it be peer development or child rearing, that stretch an already overburdened individual's time and energy (Salvagioni et al., 2017). Job burnout has been shown to be positively correlated with affective problems such as anxiety, which affects an individual's focus on children as well as developmental themes, i.e., generativity concerns (Toker and Biron, 2012).

The socio-emotional selectivity theory has been used to explain the changes in work motivation as individuals age (Carstensen et al., 1999). The theory posits that as the time horizon of a person's life course shortens, the priority of different goals changes. Individuals become more critical and devote more resources to emotionally meaningful goals and activities. In mid-adulthood, burnout leaving individuals with less energy to devote to events relevant to their sense of reproduction and, naturally, less concern for the positive aspects of themselves and the objects of their generativity. At the same time, the lack of positive feedback from generativity makes it difficult for individuals who are burned out to devote resources to people and events related to the sense of reproduction, thus reducing their attention to the generativity concern. On the other hand, the family is a social unit whose internal climate has a moderated effect on emotional stability and motivation (Miranda et al., 2000). It has been found that family members with higher family intimacy and better adaptability are less affected by the negative effects of job burnout (Voydanoff, 2002). Family members with high family intimacy and adaptability can derive emotional and material support from their family and social relationships, resulting in greater attention to things related to generativity concern (Ptacek, 1988). Therefore, this study explores the “how” and “under what conditions” burnout affects policemen's generativity concern, using subjective well-being as a mediator and family intimacy and adaptability as moderating variables.

### The Mediating Role of Subjective Well-Being Between Job Burnout and Generativity Concerns

It is a comprehensive psychological indicator of an individual's quality of life and reflects the individual's state of social functioning and adjustment (Diener et al., 1999). Conservation of resources theory suggests that individuals tend to strive for, preserve, and maintain their precious resources, which include limited time, wealth, psychological comfort, optimism, and support factors. In the workplace, when individuals perceive a threat to their resources and find it difficult to adapt to work, there is a spiral of loss of resources and eventually burnout, which undermines their happiness (Hobfoll, 2001). Research has found that burnout is an effective predictor of their subjective well-being (Cenkseven-Onder and Sari, 2009), due to the fact that individuals who are burned out severely deplete physiological and psychological resources and disrupt the work-family balance. Burnout increases an individual's unhealthy behaviors (increased unhealthy behaviors, behavioral changes such as higher smoking, lack of physical activity and sleep deprivation), which in turn leads to a variety of physiological symptoms; burnout leads to increased negative emotions and symptoms such as anxiety and depression (Genin et al., 2016); the depletion of physiological and psychological resources caused by job burnout, which in turn leads to the individual's inability to devote sufficient time and energy to other roles (e.g., family roles), and interference and conflict between different roles, which affects the individual's work-family balance (Adkins and Premeaux, 2012). Subjective well-being is closely related to generativity (Aubin and McAdams, 1995), and subjective well-being effectively predicts generativity concerns, with individuals with high life satisfaction and physical and psychological well-being paying more attention to generativity and wanting satisfaction from multifaceted developmental tasks (Hofer et al., 2008). Peterson and Duncan (2007) also found that women's satisfaction with motherhood positively predicted their generativity concern. These studies suggest that subjective well-being may mediate between job burnout and generativity concern, therefore, the current study examines the effect of job burnout, mediated by subjective well-being, on generativity concern.

### The Moderating Role of Family Intimacy and Adaptability on the Generativity Concerns of Job Burnout Effects

Family intimacy and adaptability refers to the emotional ties between family members and the ability of the family system to change in response to problems that arise in family situations and at different stages of family development (Olson, 1982). Family intimacy and adjustment have been shown to be significantly positively correlated with positive factors such as an individual's interpersonal trust (Greeff, 2000) and significantly negatively correlated with negative factors such as depression (Cumsille and Epstein, 1994). It has also been found that family intimacy is also positively correlated with adjustment and social support (Farrell and Barnes, 1993). Thus, individuals with higher family intimacy and adaptability tend to receive support and understanding from family and social relationships when experiencing job burnout, thereby reducing depression and the negative impact on reduced subjective well-being. Families with lower family intimacy and adaptability also have lower levels of trust among members. When experiencing job burnout, individuals tend to experience greater stress and negative emotions, as well as reduced generativity concerns (Serrat et al., 2017, 2018).

**Figure 1:**
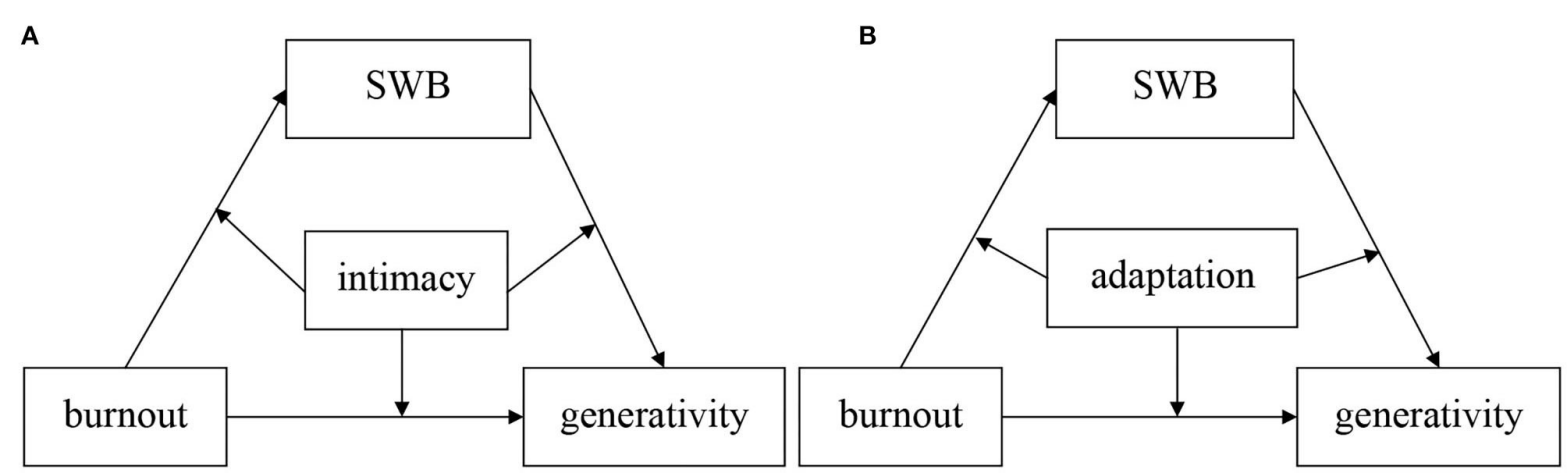
Research framework. **(A)** Regulatory role of proximity **(B)** Regulatory role of adaptation.

In summary, the moderating effects of family intimacy and adaptability may exist between job burnout and generativity concern. On the one hand, good family intimacy and adaptability can protect individuals from the negative effects of job burnout, thus reducing generativity concern; on the other hand, there is a joint effect between job burnout and low family intimacy and adaptability, thus increasing the reduction of generativity concern. Therefore, this study focuses on the local policemen to investigate the effect of job burnout on the generativity concern of individuals. An important mechanism by which job burnout affects reproductive concern is that individuals with lower subjective well-being are more likely to have negative experiences after job burnout, and thus exhibit lower generativity concern. Therefore, this study hypothesizes that H1: Subjective well-being play a mediating role between job burnout and the generativity concern of policemen. On the other hand, family intimacy and adaptability play a mitigating role in the effect of job burnout on generativity concern, and this study hypothesizes that H2: the mediating role of subjective well-being is moderated by family intimacy and adaptability, i.e., subjective well-being is a moderating mediating variable between job burnout and generativity concern, see Figure 1.

## Methods

### Participants

In this study, 550 policemen (with formal establishment, unretired) in H City from China were selected as the subjects, and 494 valid questionnaires were returned, with an effective rate of 89.8%. Among them, there were 337 males (68.2%) and 157 females (31.8%). The age of the subjects ranges from 23 to 54, with an average age of 33.71 ± 6.84.

### Measures

*The Loyola Generativity Scale (LGS)* was developed by McAdams and de St Aubin (1992). The LGS consists of 20 items, each of which is self-rated from 0 “not at all for me” to 3 “often or always for me,” with 6 items being reverse scored. In the present study, the Cronbach's alpha coefficient for the entire scale was 0.80.

*Satisfaction with life scale (SWLS)* was designed by Diener et al. (1985). The scale consists of five items scored on a 7-point Richter scale, with l indicating “strongly disagree” and 2 indicating “strongly agree.” The total score of the scale is the sum of the scores for each question, and the range of scores is from 5 to 35. Studies have shown that the scale is a valid and reliable tool for measuring life satisfaction.

*Family Intimacy and Adaptation Scale* (*FACES II*) was developed by Olson (1982). The FACES II contain 30 items, 5 of which are reverse scoring items. In this study, the Cronbach's alpha coefficients for the two subscales were 0.920 and 0.904, respectively.

*Maslach Burnout Inventory (MBI)* was developed by Maslach and Jackson (1981). The whole questionnaire consisted of 16 questions, which was later revised to 15 questions by Chinese researchers. In this study, the Cronbach's alpha coefficient for the whole questionnaire was 0.92, and the Cronbach's alpha coefficients for the three subscales were 0.94, respectively.

### Procedure

The general demographic questionnaire and the official scale were bound in a booklet, and the sampling and distribution of the questionnaires were done with the assistance of postgraduate students, using the same instructions, and collected through on-site distribution, which took ~30 min to complete. Statistical analysis of the collected data was performed using SPSS 20.0.

## Results

### Control and Testing of Common Method Deviations

The data collected in this study were collected from the self-reports of the respondents, and thus common method bias may exist. To reduce the impact of this bias on the results of the study, in terms of procedural control, this study set different response statements for each measurement questionnaire item, either in terms of degree of compliance or frequency. Statistically, the degree of common method bias of the data was tested using the Harman one-factor model method (Zhou and Long, 2004). The results showed that there were 22 factors with eigenvalues greater than one, and the first factor explained only 7.06% of the variance, which was much less than the critical value of 40%, indicating that there was no serious homoscedasticity bias for each variable in this study.

### Descriptive Statistics

The mean, standard deviation and Pearson Correlation Coefficient for each variable are presented in Table 1. The results showed that the variables of subjective well-being, generativity concern, family intimacy and adaptability were both significantly positively correlated (*r* = 0.17 ~ 0.87, *p* < 0.01) and job burnout was significantly negatively correlated (|*r* = 0.26 ~ 0.46, *p* < 0.001); and most of the correlations between demographic variables such as age, gender, with or without of child, marital status, and income, and the above-mentioned research variables have reached a statistically significant level, so it is necessary to control for them in the following analysis.

**Table 1:** Means, standard deviations, and correlations of the variables (*N* = 415).

**Variable**	**1**	**2**	**3**	**4**	**5**	**6**	**7**	**8**	**9**
1. Job burnout	–								
2. Subjective well-being	−0.46^***^	–							
3.Generativity concerns	−0.34^***^	0.57^***^	–						
4. Affinity density	−0.26^**^	0.22^***^	0.17^**^	–					
5. Adaptability	−0.28^***^	0.24^***^	0.19^***^	0.87^***^	–				
6. Ages	−0.23^***^	0.19^***^	0.14^**^	0.16^**^	0.21^***^	–			
7. Gender	−0.07	0.14^**^	0.13^*^	0.03	0.04	0.03	–		
8. With child	−0.15^**^	0.10^*^	0.12^*^	0.22^***^	0.17^**^	0.50^***^	0.11^*^	–	
9. Marital status	−0.14^**^	0.09	0.06	0.32^***^	0.26^***^	0.34^***^	0.10^*^	0.65^***^	–
10. Income	−0.19^***^	0.27^***^	0.27^***^	0.17^**^	0.16^**^	0.32^***^	0.10^*^	0.18^**^	0.18^**^
*M*	3.04	2.70	2.12	63.80	51.81				
*SD*	0.96	0.44	0.41	10.60	10.41				

*Note*:

* *p < 0.05*,

** *p < 0.01*,

*** *p < 0.001*.

### Test for Moderated Mediation Effect

Following the recommendations of Muller et al. (2005), testing a moderated mediating model requires the estimation of three equations: Equation 1 estimates the moderating effect of the moderating variable (intimacy) on the relationship between the independent variable (job burnout) and the dependent variable (generativity concern). Equation 2 estimates the moderating effect of the moderating variable (intimacy) on the relationship between the independent variable (job burnout) and the mediating variable (subjective well-being). Equation 3 estimates the moderating effect of the moderating variable (intimacy) on the relationship between the mediating variable (subjective well-being) and the dependent variable (generativity concern). All predictor variables were standardized in each equation, controlling for demographic variables such as age and gender.

As shown in Table 2, job burnout significantly negatively predicted generativity concern in Equation 1 (*b* = −0.259, *p* < 0.001), but the interaction term for job burnout and intimacy was not a significant predictor of generativity concern (*b* = −0.002, *p* > 0.05). Job burnout negatively predicted subjective well-being in Equation 2 (*b* = −0.388, *p* < 0.001), but the interaction term for job burnout and intimacy was not significant in predicting subjective well-being (*b* = 0.075, *p* > *0*.01). Subjective well-being in Equation 3 positively predicted generativity concern (*b* = 0.467, *p* < 0.001), while the interaction terms of subjective well-being and intimacy were also significant in predicting generativity concern (*b* = 0.220, *p* < 0.001). Thus, the four variables of job burnout, subjective well-being, intimacy, and generativity concern constituted a moderated mediation model, and the moderating effect occurred only in the second half of the mediating effect, i.e., the effect of subjective well-being on generativity concern was moderated by intimacy.

**Table 2:** Moderated mediation effects test for intimacy.

**Predictive variables**	**Equation 1: Generativity concern**			**Equation 2: Subjective well-being**			**Equation 3: Generativity concern**		
	***b***	***SE***	***t***	***b***	***SE***	***t***	***b***	***SE***	***t***
Age	−0.002	0.006	−0.350	0.008	0.006	1.347	−0.006	0.005	−1.132
Genders	0.097	0.108	0.893	0.164	0.102	1.609	0.060	0.095	0.626
With or without child	0.214	0.142	1.514	−0.026	0.133	−0.193	0.274	0.124	2.215^*^
Marital status	−0.233	0.136	−1.718	−0.125	0.128	−0.980	−0.221	0.119	−1.859
Earnings	0.429	0.107	3.994^***^	0.346	0.101	3.421^***^	0.276	0.095	2.900^***^
Work burnout	−0.259	0.052	−0.015^***^	−0.388	0.049	−0.974^***^	−0.088	0.049	−1.797
Intimacy	0.076	0.056	1.373	0.063	0.052	1.205	0.033	0.049	0.686
Job burnout × intimacy	−0.002	0.044	−0.048	0.075	0.042	1.798	0.072	0.046	1.567
Subjective well-being							0.467	0.050	9.338^***^
Subjective well-being × intimacy							0.220	0.050	4.381^***^
*R*^2^	0.145			0.241			0.354		
*F*	8.390^***^			14.870^***^			20.133^***^		

*Note*:

* *p < 0.05*,

** *p < 0.01*,

*** *p < 0.001*.

**Figure 2:**
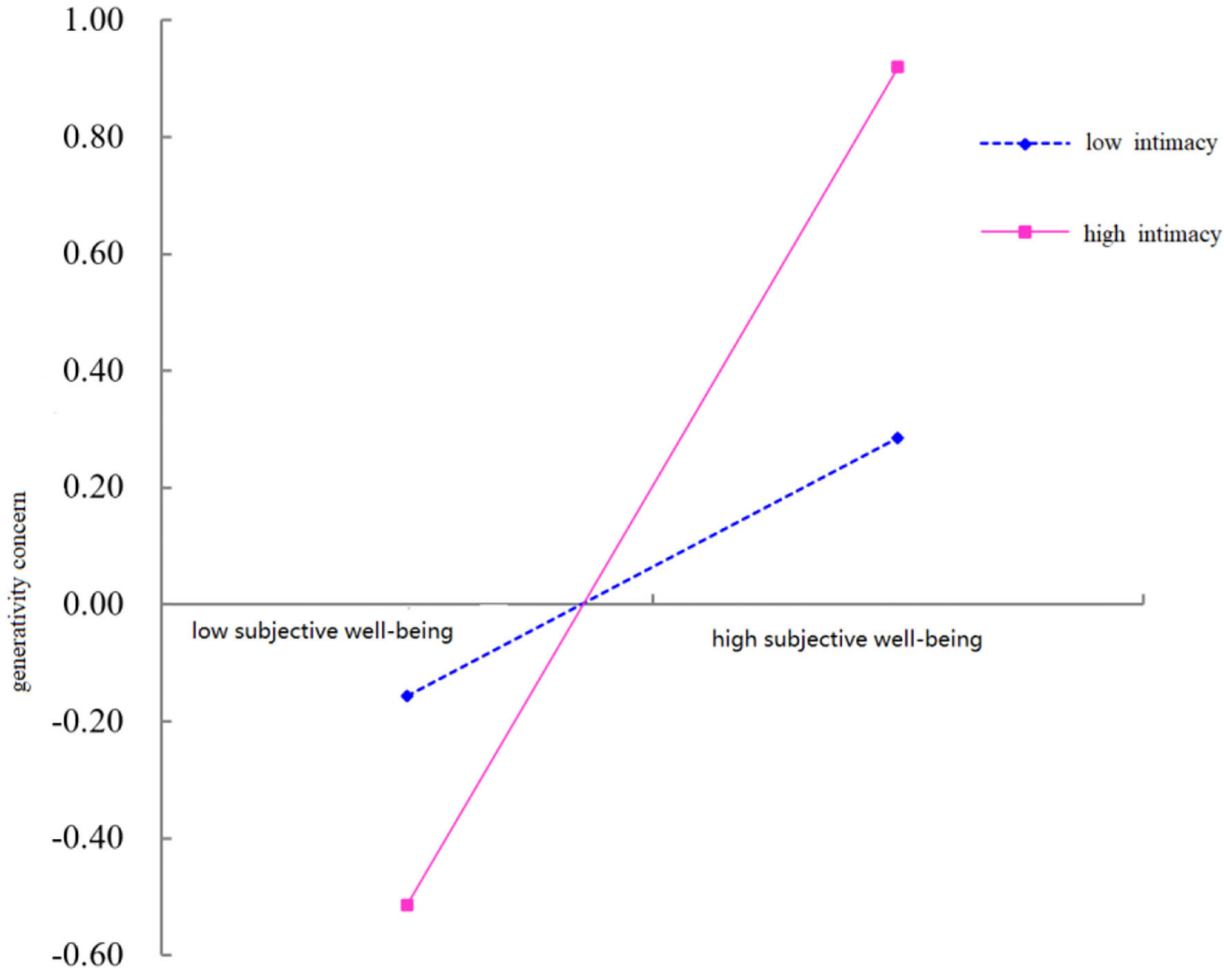
The moderating effect of intimacy on the relationship between subjective well-being and generativity concerns.

To reveal how intimacy modulates the effect of subjective well-being on generativity concern, we grouped high and low according to the score of intimacy and plotted the interaction as shown in Figure 2. The simple slope test (simple slope test) showed that when the level of intimacy was high (+1), the degree of subjective well-being on generativity concern showed a more obvious upward trend (*b* = 0.687, *t* = 10.862); when the level of intimacy was low (−1), the degree of subjective well-being on generativity concern showed an upward trend with a signs of leveling off (*b* = 0.247, *t* = 3.905). Thus, intimacy can play a facilitating role in the positive predictive effect of subjective well-being on generativity concerns.

The same procedure was used to test for the moderating effect of adaptation. As shown in Table 3, job burnout significantly negatively predicted generativity concern in Equation 1 (*b* = −0.250, *p* < 0.001), but the interaction term of job burnout and adaptability was not a significant predictor of generativity concern (*b* = 0.007, *p* > 0.05). Job burnout negatively predicted subjective well-being in Equation 2 (*b* = −0.385, *p* < 0.001), but the interaction term for job burnout and adaptability was not significant in predicting subjective well-being (*b* = 0.093, *p* > 0.05). In Equation 3 subjective well-being positively predicted generativity concern (*b* = 0.469, *p* < 0.001), while the interaction term of subjective well-being and adaptability was also significant in predicting generativity concern (*b* = 0.248, *p* < 0.001). Thus, the four variables of job burnout, subjective well-being, adaptability, and generativity concern also constituted a moderated mediation model, and the moderating effect occurred only in the second half of the mediation effect, i.e., the effect of subjective well-being on generativity concern was moderated by adaptability.

**Table 3:** Moderated mediated effects test for adaptation.

**Predictive variables**	**Equation 1: Generativity sense concern**			**Equation: 2: Subjective well-being**			**Equation 3: Generativity sense concern**		
	***b***	***SE***	***t***	***b***	***SE***	***t***	***b***	***SE***	***t***
Age	-0.003	0.006	-0.543	0.007	0.006	1.326	-0.008	0.005	-1.449
Genders	0.104	0.108	0.963	0.151	0.102	1.478	0.073	0.094	0.776
With or without children	0.227	0.141	1.607	-0.014	0.133	-0.108	0.267	0.123	2.172^*^
Marital status	-0.242	0.134	-1.801	-0.117	0.126	-0.924	-0.214	0.117	-1.840
Earnings	0.432	0.107	4.023^***^	0.353	0.101	3.486^***^	0.276	0.095	2.911^**^
Work burnout	-0.250	0.052	-4.813^***^	-0.385	0.049	-7.859^***^	-0.088	0.049	-1.786
Adaptive	0.117	0.057	2.058^*^	0.070	0.054	1.307	0.069	0.050	1.399
Job burnout × adaptability	0.007	0.046	0.144	0.093	0.043	2.153^*^	0.083	0.048	1.751
Subjective well-being							0.469	0.050	9.388^***^
Subjective well-being × adaptability							0.248	0.053	4.652^***^
*R^2^*	0.151			0.242			0.363		
*F*	8.760^***^			14.904^***^			20.844^***^		

*Note*:

* *p < 0.05*,

** *p < 0.01*,

*** *p < 001*.

**Figure 3:**
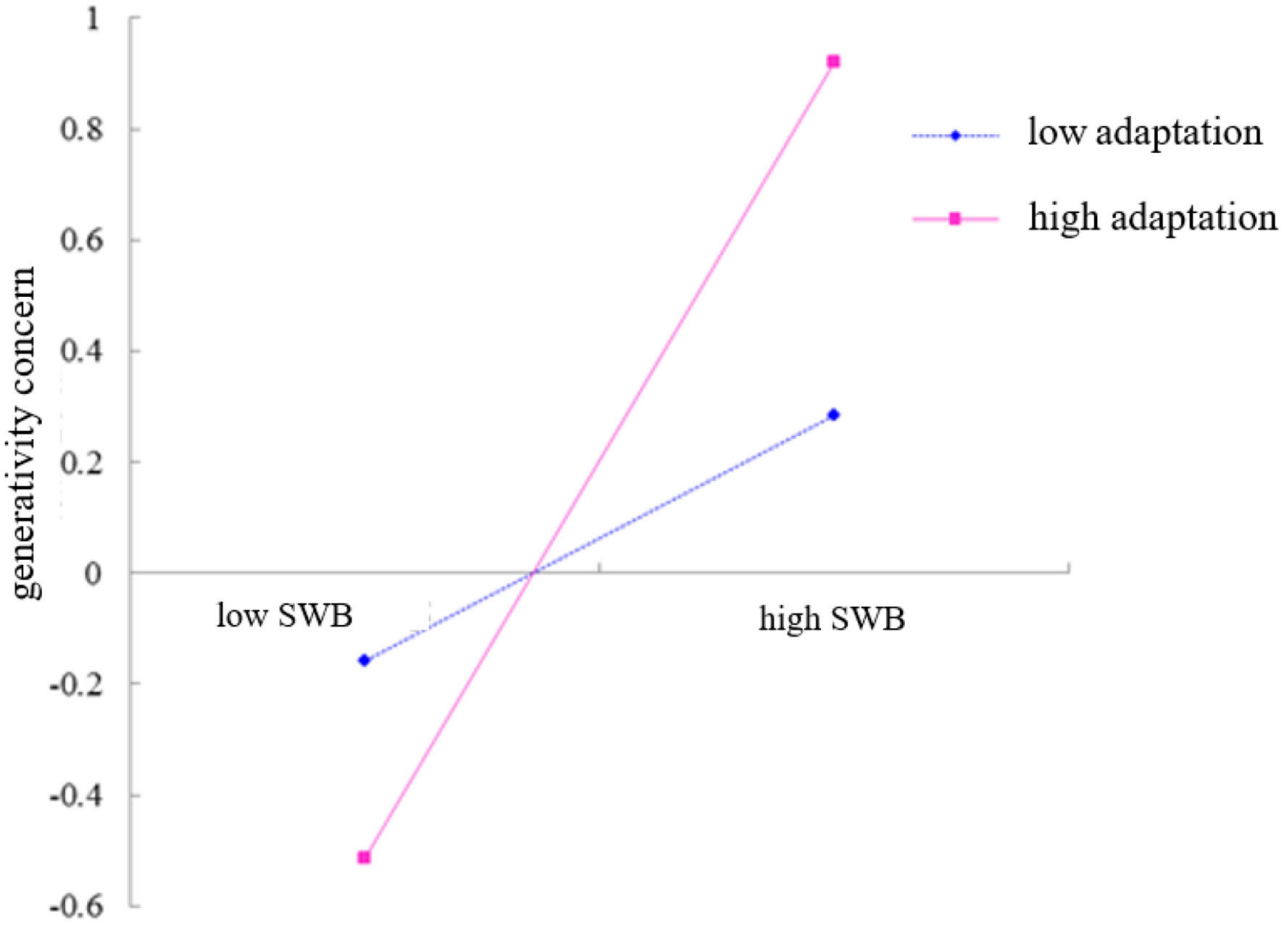
The moderating effect of adaptation on the relationship between subjective well-being and generativity concern.

To reveal how adaptation modulates the effect of subjective well-being on generativity concern, the interaction diagrams shown in Figure 3 are plotted by grouping high and low according to the score of adaptation. A simple slope test showed that when the level of adaptation was high (+1), the degree of influence of subjective well-being on generativity concern showed a more pronounced upward trend (*b* = 0.717, *t* = 11.337); when the level of adaptation was low (−1), the upward trend of the degree of influence of subjective well-being on generativity concern showed signs of flattening (*b* = 0.221, *t* = 3.494). Thus, adaptation can play a facilitating role in the positive predictive role of subjective well-being on generativity concerns.

## Discussion

This study found that job burnout negatively influenced generativity concern, which is consistent with previous findings that the higher the level of job burnout, the lower the generativity concern (Berkowitz, 1987).

The current findings also show that subjective well-being mediates between job burnout and generativity concern, which confirms our hypothesis. Poor wellbeing and moderate to high levels of burnout are associated (Hall et al., 2016). Adults' satisfaction in work and life can extend to a broader range of concerns, such as concern for their own achievements, concern for the growth and well-being of the next generation, caring for others, and caring for the community, i.e., generativity concern (Hofer et al., 2008). When job burnout occurs, adults' physiological and psychological resource depletion and work-family balance are disrupted, which negatively affects an individual's subjective well-being and may even produce anxiety and depression (Judge and Bono, 2001; Schwarzer and Hallum, 2008; Kowalczuk et al., 2020), all of which are detrimental to an individual's of productive and creative activities. Adult subjective well-being is closely related to generativity concern, and a decrease in well-being affects an individual's generativity concern, i.e., a decrease in concern, care, and concern for the next generation, others, and society (Serrat et al., 2017, 2018). The current study provides supportive evidence for this hypothesis.

The present study found that there was a moderating effect of family intimacy and adaptability on the indirect effect between job burnout and generativity concern. Work-family conflict can predict greater levels of emotional exhaustion, the multi-group path models test suggest that the type and level of job resource moderates the relationship between job burnout and job satisfaction (Lizano and Mor Barak, 2015). For different levels of family intimacy and adaptability, the magnitude of the mediating effect of job burnout through subjective well-being on generativity sense concern varied. The indirect effect was more pronounced for adults with lower family intimacy and adaptation than for adults with higher family intimacy and adaptation. Specifically, the moderating effect of family intimacy and adaptability occurs in the second half of the mediating chain pathway, where the relationship between subjective well-being and generativity concern depends on the level of family intimacy and adaptability. As shown in Figures 2, 3, low levels of family intimacy and adaptability can significantly reduce the positive effect of subjective well-being on generativity concern, while high levels of family intimacy and adaptability can enhance this effect. Thus, for adults who are burned out, low levels of family intimacy and adaptability and high levels of family intimacy and adaptability play the roles of “adding insult to injury” and “give timely assistance,” respectively.

Family intimacy and adaptability, as important family relationship indicators that measure the degree of emotional and material support available to adults in the family environment (Cumsille and Epstein, 1994), play an extremely important role in adults' social relationships. Good family intimacy and adaptability allows family members to enjoy a warm and supportive family environment, prompting the psychological experience of their productive and creative activities to extend into a wider space, resulting in a higher willingness to care for others and for the community. The psychological processes resulting from subjective well-being can be weakened or enhanced in the important environment of the family.

## Conclusion

Subjective well-being mediates between burnout and generativity concern, i.e., burnout has an indirect effect on generativity concern by influencing the subjective well-being of adults.

The relationship between subjective well-being and generativity concern (the second half of the mediation effect) is moderated by family intimacy and adaptation, both of which enhance the positive effect of subjective well-being on generativity concern.

## Study Limitations

Though this study makes several contributions to the literature on job burnout and affective well-being, its limitation is worthy of note. The sample used, study design (cross-sectional study), lack of adjustment for possible confounders and was conducted only in H city from China are significant limitations of the study. In using an availability sample of police officers, there is a risk of potentially having a biased sample.

## Data Availability Statement

The raw data supporting the conclusions of this article will be made available by the authors, without undue reservation.

## Ethics Statement

The studies involving human participants were reviewed and approved by The study protocol was approved by Hezhou University Ethnics Committee, and has been executed consistent with ethical standards laid down in the 1964 Declaration of Helsinki and its later amendments. The patients/participants provided their written informed consent to participate in this study.

## Author Contributions

XL and YL designed the study. XL, GW, and HY collected data, developed, and performed the statistical analysis in conjunction. XL and YL wrote the first draft of the manuscript. All authors contributed to the article and approved the submitted version.

## Conflict of Interest

The authors declare that the research was conducted in the absence of any commercial or financial relationships that could be construed as a potential conflict of interest.
